# Preparation, Biological Activities, and Potential Applications of Hen Egg-Derived Peptides: A Review

**DOI:** 10.3390/foods13060885

**Published:** 2024-03-14

**Authors:** Li Song, Yi Chen, Huiping Liu, Xiaowei Zhang

**Affiliations:** Tianjin Key Laboratory of Food Quality and Health, College of Food Science and Engineering, Tianjin University of Science & Technology, Tianjin 300457, China; songli20200505@163.com (L.S.); 16622881022@163.com (Y.C.); liuhuiping@tust.edu.cn (H.L.)

**Keywords:** egg-derived peptides, enzyme inhibitors, intestinal health, co-delivery system

## Abstract

Food-derived peptides have been extensively studied for their benefits in humans. Hen eggs, characterized by high protein and digestibility, are an excellent source of food-derived bioactive peptides. This review summarizes the preparation methods, purification, and identification of hen egg-derived peptides (HEPs). The preparation methods mainly include enzymatic hydrolysis, microbial fermentation, and chemical synthesis. Genetic engineering is an emerging trend of HEP preparation. Then, we summarize the biological activities of HEPs, such as antioxidant activities, enzyme inhibitory activity, and antibacterial activity, of which the enzyme inhibitory activity is comprehensively summarized for the first time. The structure–activity relationship and underlying mechanism of the HEPs are further elucidated. Finally, the applications, future challenges, and opportunities of HEPs were mainly discussed in the food and non-food sectors. We focus on the potential applications of HEPs in intestinal health and assembly delivery and provide a reference for the further utilization and commercial development of HEPs.

## 1. Introduction

Hen eggs are a high-demand food on the global market. In 2019–2021, the average annual production of hen eggs reached 90,513 million tons and was consumed at a rate of 10.6 kg per capita per year [1]. Hen eggs have been described as near-perfect because of their nutritional balance, high digestibility, and affordable price [2]. Proteins are the primary nutrient in hen eggs. Hen egg proteins have high digestibility and absorption rates, and are called complete proteins [3]. Proteomic analyses identified 371, 428, and more than 500 proteins in egg white, yolk, and eggshell membrane proteins, respectively [4,5]. Egg white proteins mainly include ovalbumin, ovotransferrin, ovomucin, ovomucoid, and lysozyme [6]. Egg yolk proteins mainly include lipovitellin, globular protein, phosvitin, and low-density lipoprotein [7]. Eggshell membrane proteins mainly include collagen, keratin, agrin, and laminin [8]. Hen egg proteins possess many functional activities, including antibacterial, immunomodulatory, and antioxidant [9,10,11]. In particular, the antioxidant activity has attracted considerable attention. Moreover, ovalbumin, ovotransferrin, and lysozyme often exhibit significant antioxidant properties [10]. After different enzyme hydrolysis, hen egg proteins generate bioactive peptides that demonstrate significantly enhanced biological activities compared to the original proteins, or even new biological activities. Yeon Cho et al. (2023) found that enzymatic hydrolysis improved the nutritional and functional characteristics of the proteins [12]. Studies have shown that peptides exhibit the higher absorptivity and carrier saturation in recent years than proteins [13]. They are easily absorbed by enterocytes in the small intestine and enter the portal vein through capillary vessels in the small intestine [14]. Some peptides comprising fewer than six amino acids may resist gastrointestinal digestion and remain intact as they cross the intestinal epithelium [15]. Animal and plant proteins are the primary sources of food-derived bioactive peptides. However, bioactive peptides derived from plants are always deficient in one or more essential amino acids, leading to a reduced nutritional value and bioavailability [16]. An increasing interest has been shown to bioactive peptides of animal origin, particularly milk and egg origins. Hen eggs contain various proteins and provide a comprehensive nutrition for the development of hen embryos [17]. Therefore, an increasing body of research suggests that hen egg-derived peptides (HEPs) may exhibit a superior biological activity than their protein counterparts in many cases [18]. For example, phosvitin (PV) has a strong binding affinity to iron, making it difficult to release its ion, which results in a decreased intestinal absorption [19]. Feng et al. (2005) proposed that phosvitin phosphopeptides (PPPs), obtained by the enzymatic hydrolysis of PV, could promote absorption and bioavailability in the monolayer model of Caco-2 cells [20]. Moreover, ovomucin (OM) does not possess antioxidant activity, whereas the hydrolysates exhibit more [21]. Ibrahim et al. (2001) confirmed that the antibacterial activity of lysozymes improved greatly after enzymatic hydrolysis, attributed to the exposure of the antibacterial active sites of the protein [22]. Beyond that, HEPs possess a range of regulatory properties, such as angiotensin-I-converting enzyme (ACE) inhibitory, immunomodulatory, and anti-inflammatory activities [23,24]. Recent reviews have summarized the preparation of HEPs from a specific part of the egg, as well as certain biological properties and potential applications in the food and pharmaceutical industries. Moreover, there are few summaries of HEPs based on preparation methods, and a limited discussion of the potential applications and biological activities. This article reviews the preparation using different methods to obtain HEPs. It also offers a comprehensive summary of the main biological activities and reviews the enzyme inhibition activity of HEPs for the first time. Finally, we summarize the potential applications of HEPs in improving intestinal health and assembly delivery in both the food and non-food sectors.

## 2. Bioactive Peptides Derived from Hen Egg Proteins

The eggshell membrane (ESM) is a polymeric fibrous meshwork, and the primary component is protein, which accounts for approximately 90% [24] ESM proteins mainly consist of collagen, keratin, agrin, and laminin [8]. However, the eggshell membrane is difficult to separate from hen eggs [25]. Microwave-assisted separation, chemical separation, and vacuum treatment are widely used to separate eggshell membranes [4,26,27]. Moreover, it has been reported that hydrolyzed ESM are used more widely than ESM, improving the bioavailability of the active components [4]. The main component of the whole egg is egg white, accounting for 63% of the whole egg weight, of which the content of protein is 10.5% [28]. Egg white proteins (EWPs) mainly contain ovalbumin (OVA, 54%), ovotransferrin (OTF, 12%), OM (11%), ovomucin (OV, 3.5%), lysozyme (LM, 3.4%), globulin (8%), and avidin (0.05%) [28]. Therefore, EWPs have a high nutritional value, and its hydrolysates have various biological properties after hydrolysis. The egg yolk has 16% proteins, mainly including low-density lipoprotein, high-density lipoprotein, livetins (α, β, and γ), and PV [29]. PV and gamma-livetin are the most studied egg yolk proteins. Gamma-livetin mainly consists of immunoglobulin Y (Ig Y), which can react with serum immunoglobulins. Because it is relatively easy to obtain and purify, it is used as a substitute of mammalian antibodies [30]. In particular, yolkin is a polypeptide complex accompanied by Ig Y, consisting of several peptides and proteins [31]. PV is a highly phosphorylated protein with excellent emulsification, antioxidant, and metal-chelating properties, which has a great application potential [32].

EWP, OVA, OVTF, OM, LM, PV, and ESM proteins have most commonly been used as precursor proteins. HEPs are mainly prepared via enzymatic hydrolysis, microbial fermentation, chemical synthesis, and chemical hydrolysis. However, after chemical hydrolysis, the resulting molecules may become non-absorbable and have poor nutritional qualities or low functionalities [33]. Therefore, there is little research on the preparation of HEPs using chemical hydrolysis. 

### 2.1. Peptide Preparation

#### 2.1.1. Enzymatic Hydrolysis

Enzymatic hydrolysis is a process that involves the cleaving of protein structures using proteases, thereby releasing bioactive peptides [34]. The enzyme type and reaction conditions play a vital role in the preparation of HEPs, determining the composition and sequence of amino acids [35]. There are two main ways to utilize enzymatic hydrolysis for the preparation of bioactive peptides: in vivo gastrointestinal digestion and in vitro enzymatic hydrolysis. In vivo gastrointestinal digestion entails the proteins entering the human body orally and being degraded by gastrointestinal (GI) enzymes, such as pepsin, trypsin, and chymotrypsin. However, only some studies have focused on the preparation of HEPs by in vivo gastrointestinal digestion due to the complexity of organisms and the limitations of genotype information. Currently, most studies have prepared HEPs by in vitro simulated GI digestion [36]. Zhang et al. (2023) reported that EWPs were subjected to in vitro gastrointestinal digestion simulation using pepsin and trypsin, and found that EWP peptides (EWPP) were released abundantly in the stomach, with 307 peptides being released in the stomach and 160 peptides released in the intestine [37]. Palika et al. (2015) reported that food peptides, released during the in vitro gastrointestinal digestion of hen egg whites, could bind and solubilize ferric iron [38]. In vitro enzymatic hydrolysis entails bioactive peptides being prepared by non-GI proteases derived from plants or microorganisms. The proteases commonly used for this purpose include bromelain, papain, alcalase, thermolysin (EC 3.4.24.27), and neutral proteases (EC 3.4.24.4) [36,37].

Moreover, the biological activities of peptides obtained from some non-GI proteases are stronger than those from GI protease hydrolysis, due to differences in the hydrolysis specificity. Young et al. (2010) presented a comparison of the phosphate content of egg yolk peptides (EYP) from alcalase (EC 3.4.21.62) and protease S, protease N (EC 3.4.24.28), protease A (EC 3.4.24.39), protease P, and trypsin (EC 3.4.21.4) digestion, and found that EYPs from protease P had the highest phosphate content (43.95 μg of PO_4_/mg of protein), thus exhibiting a higher antioxidant stress bioactivity [39]. Five proteases (alcalase, trypsin, neutrase (232-752-2), papain, and pepsin (EC 3.4.23.1)) were used as hydrolytic enzymes to produce antioxidative peptides from ESMs, and found that the highest free radical scavenging activity belonged to alcalase hydrolysates (degree of hydrolysis, DH of about 28%) [25]. Abeyrathne et al. (2016) evaluated the inhibition of ACE by ovomucin-derived peptides (OMPs) obtained using trypsin, papain, and alcalase under their optimum conditions, and found that OMPs from alcalase and papain (EC 3.4.22.2) exhibited the highest ACE inhibitory activity (>70%) [21]. Alcalase mainly acts on the peptide bonds of Ser, Thr, Asp, and Glu. Papain cleaves the peptide bonds of amino acids, including Leu, Gly, and Cys. Trypsin breaks down peptide bonds containing Lys and Arg. Memarpoor-Yazdi et al. (2012) proposed that the antioxidant activity of egg white lysozyme peptides (LMPs) obtained from papain were better than that of trypsin. The DPPH and ABTS radical scavenging activities of the LMPs were 37.2 ± 1.69%, 50.4 ± 2.1%, 1.91 ± 0.13, and 2.57 ± 0.19 μmol Trolox equivalents (TE)/mg protein for trypsin and papain, respectively [40].

ESMs have been hydrolyzed by alkaline protease from *Bacillus altitudinis* GVC11, resulting in protein hydrolysis with a significant antioxidant activity [41]. The enzyme produced by the Pseudomonas aeruginosa strain ME-4 can hydrolyze the ESM and produce two water-soluble peptides containing proline and the free amino acid tryptophan [42].

Furthermore, the biological activity of peptides is related to the length of the amino acid sequence. The DH and peptide chain length of HEPs are improved by the hydrolysis of two or more enzymes. Currently, there are two critical parameters used to assess the extent of enzymatic proteolysis: the DH and molecular weight distribution (MWD) of peptides. Thus, the combination of different enzymes may be another viable option. Shi et al. (2014) prepared ESMP with higher antioxidant activity by single or the combination of enzymatic hydrolysis (alcalase, protease A, protease N, protease P, and protease S); the DH and total nitrogen recovery from combinatorial enzymatic hydrolysis were higher than those from single enzymatic hydrolysis, 26.39 ± 0.46% (the hydrolysate of alcalase and protease P) and 65.60 ± 0.43% (the hydrolysate of alcalase and protease S), respectively [43]. Then, three groups of peptides with different molecular weights, isolated and purified from the hydrolysate of alcalase and protease S, showed excellent antioxidant activity, which improved the cellular redox state of Caco-2 cells by inhibiting IL-8 secretion and increasing the GSH concentration [43]. OVAPs (ovalbumin-derived peptides) were obtained by hydrolysis with 1% of pepsin, trypsin, α-chymotrypsin (EC 3.4.21.1), papain, and alcalase singly or in combination at 37 °C, for 4 h, and found that the Fe-chelating capacity of the hydrolysates differed among the enzyme treatments: the alcalase + trypsin and chymotrypsin (232-671-2) treatment was the best, followed by the pepsin +papain and pepsin + alcalase treatments [44]. Papain, alcalase, trypsin, and thermolysin are widely used to prepare HEPs. At present, HEPs mainly act as functional additives or functional drinks. For instance, Zheng et al. (2020) proposed that HEPs could be used to prepare peptide with hypotensive effects [45] EWPPs could act as antioxidants and anti-inflammatory additives to be applied in food and drugs [46]. Moreover, HEPs were also incorporated into a functional beverage to enhance the nutritional value of foods [47].

Currently, enzymatic hydrolysis is the most common method used to generate HEPs. It is milder than other methods, and the obtained proportions of some essential amino acids were higher than those in previous studies [48]. HEPs, obtained through proteolysis by food-grade commercial enzymes, have been generally regarded as safe (GRAS). However, conventional enzymatic hydrolysis has a low efficiency because of the infrequent contact between the substrate and enzyme. Thus, studies have reported that some assistive technologies or pretreatments can be applied to enhance the efficiency and bioactive peptide yield, including pulsed electric field, ultrasound treatment, thermal denaturation, and high-pressure treatment [49]. Among them, the preparation of PPPs is dramatically affected by enzymatic hydrolysis. Early studies have shown that PV containing phosphorylated amino acid residues showed an antagonistic effect on the protease activity, significantly reducing the efficiency of enzymatic hydrolysis [50]. In order to improve the efficiency of enzymatic hydrolysis, some auxiliary technologies have been used as pretreatment, such as NaOH treatment, phosphatase treatment, heat treatment, and pressure treatment [51]. Jiang et al. (2000) applied the alkali treatment method using various concentrations of a NaOH solution for the dephosphorylation of PV, resulting in an improved DH [52]. Samaraweera et al. (2013) reported that acid or alkali pretreatments could improve the hydrolysis process of PV, thereby enhancing its functionality [53]. Moreover, proteases were used to hydrolyze the high-pressure (600 MPa) and phosphatase-pretreated PV to obtain a relatively large molecular weight of peptides [54]. Huang et al. (2019) reported that high-temperature and mild-pressure (HTMP, at 100 °C for 60 min) pretreatment could help the hydrolysis of PV further, and the DH values of enzyme combinations (trypsin and multifect 14 L) were the highest compared to those of other combinations, with a DH of 26.01 ± 1.23% [55]. PV dephosphorylated by alkaline phosphatase (E.C.3.1.3.1) was digested using trypsin, pepsin, or thermolysin (EC 3.4.24.27), and low-molecular-weight peptides (<5 kDa) in trypsin hydrolysates and alkaline hydrolysis accounted for 23.59% and 21.22%, respectively [56]. The enzymatic hydrolysis of other proteins in eggs can be significantly enhanced by assistive technologies. Jain et al. (2016) found that ESMPs, prepared through a combination of ultrasound enzymatic hydrolysis (alcalase and papain), exhibited superior physicochemical properties (emulsifying properties, foaming properties, and water-holding capacity) than conventional enzymatic hydrolysis, and the optimal preparation conditions were amplitude, time, and solid-to-solvent ratio values of 95.74%, 28.06 min, and 1:30 (g: mL), respectively [57]. Lei et al. (2011) demonstrated that ultrasound pretreatment could increase the reactive sulfur groups in a 5% ovotransferrin solution by 50% [58]. Shen et al. (2010) also reported that ovotransferrin-derived peptides (OTFPs) were obtained using ultrasound pretreatment-assisted thermolysin hydrolysis, whose antioxidant value was further increased from 0.49 to 1.95 μmol of TE/mg [59]. This might be because microwave energy could prevent protein aggregation to improve enzymatic hydrolysis by penetrating inside the samples. In addition, pulsed electric field (PEF) is a green pretreatment technology with a low energy consumption and high efficiency, which is widely applied in multiple domains. Li et al. (2023) found that PEF-assisted alcalase hydrolysis increased the DH (46.81%), surface hydrophobicity (decreased by 13.60%), and free sulfhydryl group content (increased by 79.85%) [60]. Liu et al. (2018) reported that the PEF treatment (653–695 kJ/kg, 1.4–1.7 kV/cm) had an enhancing effect on the enzymatic hydrolysis of ovomucin. However, a significant enhancement only arose from the PEF treatment (695 kJ/kg, 1.7 kV/cm) of ovomucin at acidic (pH 4) conditions, which showed a similar DH compared to ovomucin heat-treated at 80 °C [61]. This can be due to the fact that the PEF treatment may alter the conformational space of proteins to expand the secondary and tertiary structures. Thus, the optimization of these factors, such as the protease type, hydrolysis conditions, and other factors, is effective in obtaining target bioactive peptides.

#### 2.1.2. Microbial Fermentation

Microbial fermentation is a method that catalyzes the preparation of HEPs under specific conditions using enzymes derived from certain microorganisms. Microbial fermentation produces a complex array of enzymes to form a complex enzyme system. Microbial fermentation has the advantage of relatively low production costs, but it has the disadvantages of a low peptide yield and a lack of specificity in peptide formation [62]. Lactic acid bacteria (LAB) fermentation is the simplest and safest technology, which affects the protein structure by enzymatic action and acidification. Nahariah et al. (2020) reported that Lactobacillus sp (LAB), consisting of *L.bulgaricus, L.achidopillus*, and Streptococcus thermophilous, egg proteins were used in egg protein fermentation to obtain HEPs with antioxidant activity [63]. PPPs were prepared by lactic acid bacteria (*S. thermophilus: L. bulgaricus* 1:1) fermentation at 1% *w*/*w* PV, 42 °C, for 9 h, and found that its calcium-binding capacity (NaOH consumption of 0.9 mL) was enhanced compared to the PPPs produced by enzymatic digestion (NaOH consumption of 2.85 mL) [64]. In another study, ESMPs were hydrolyzed by *Lactobacillus plantarum* fermentation under the optimal environmental conditions (pH 8.0 and 36 h), resulting in the maximum protein concentration (177.3 mg/g) and DH (25.1%) of the hydrolysates [65]. In addition, during fermentation, LAB can produce a little lipase to be applied in fat breakdown. Proteases derived from LAB are commonly used in the preparation of bioactive peptides that act as natural feed additives in animal nutrition. However, microbial metabolites are complex and diverse, making the isolation and purification of peptides difficult. Therefore, further research is necessary to address the issues.

#### 2.1.3. Chemical Synthesis

The chemical synthesis method entails preparing bioactive peptides based on known amino acid sequences, and the commonly used chemical synthesis includes liquid-phase synthesis and solid-phase synthesis (SSPS). SPSS is one of the most common techniques used in peptide synthesis, and the general process involves adding amino acids one by one from the C-terminus to the N-terminus of the sequence to synthesize the target peptides [66]. Moreover, SPPS is the preferred method for synthesizing peptides in laboratories and the industry due to its simplicity, effectiveness, and characteristics of automated synthesis. However, the amino acid sequence of the target peptide is essential for synthesizing peptides. Thus, most studies combine enzymatic digestion and SSPS, and the general process is: HEPs are prepared by protease; then, the peptides of simple components are identified by isolation and purification, and finally, the target bioactive peptide is synthesized according to the requirements. For instance, Liu et al. (2015) reported that the hydrolysates of egg white proteins were isolated and purified to synthesize EWPPs (DHTKE, FFGFN, and MPDAHL) by SSPS [11]. Zhang et al. (2021) isolated and solid-phase synthesized a potent calcium-binding peptide (DEEENDQVK) from the high-phosphorus protein hydrolysate of yolks (calcium-binding capacity increase to 151.1 mg/g) [67]. Si et al. (2023) also reported that PPPs with a high calcium-binding capacity were identified and synthesized from yolk high phosphoprotein hydrolysates (Glu-Asp-Asp-pSer-pSer) and found that the maximum calcium-binding capacity of EDDpSpS was 468 ± 2.80 mg/g [32]. However, egg-derived synthetic peptides are mainly used for laboratory experiments. For instance, Sun et al. (2017) investigated the binding mechanism of Asp-His-Thr-Lys-Glu (DHTKE) from egg whites to calcium and the calcium absorption of a peptide–calcium complex by a Caco-2 cell model, and found that this complex increased calcium absorption capacity by 7 times [68]. And the KPHAEVVLR (KR-9) peptide, derived from hydrolyzed egg whites, was reported to accelerate the wound healing of the palatal mucosa in rats by promoting human gingival fibroblast proliferation [69].

The various methods of preparing HEPs discussed in this review are summarized in Table 1. Upon reviewing the literature, we found that enzymatic hydrolysis and SSPS are widely used in the preparation of HEPs. Enzymatic hydrolysis is a mature method with high safety, specificity, and activity, making it suitable for production. The low content and purity of HEPs obtained from enzymatic hydrolysis do not contribute to the application of their related products. SSPS has the advantages of a short synthesis cycle and high production, which can effectively overcome the deficiency of natural HEPs. However, synthesis is only suitable in laboratories, due to its low yield and high cost. Beyond that, genetic engineering strategies for the production of peptides have been a research hotspot. Feng et al. (2010) reported that the bovine lactoferricin derivative peptide LfcinB-W10 was successfully expressed by *Escherichia coli*, and found that it exhibited growth inhibition activity against *Staphylococcus aureus* ATCC25923 [70]. At present, some studies have reported that some hen egg proteins can be successfully expressed using genetic engineering strategies. For instance, with the assistance of a xylanase fusion partner, hen egg-white lysozymes were recombinantly expressed in *Pichia pastoris* [71]. Therefore, a new research trend in the preparation of HEPs is genetic engineering strategies.

### 2.2. Purification and Identification of Peptides

Enzymatic hydrolysis has the disadvantages of high cost, low peptide yield, and difficult separation and purification of the mixed peptides. Microorganisms are relatively inexpensive sources of proteases, and microbial fermentation uses proteases that can be produced by microbial strains to hydrolyze substrate proteins. HEPs are a complex mixture, mainly containing hen egg proteins, peptides of various molecular weights, free amino acids, and salts. To further investigate the biological properties and the structure–activity relationship, it is necessary to obtain highly purified/single fractions of HEPs. The methods for the isolation and purification of peptides are mainly membrane separation, electrophoresis, and chromatography [51]. Moreover, the purification scheme for HEPs consists of multiple combined purification procedures, including ion-exchange chromatography, gel filtration chromatography, affinity chromatography, and reverse phase-high-performance liquid chromatography (RP-HPLC). Sun et al. (2014) reported that EWPPs fractions with different molecular weights (>5, 2–5, 1–2, and <1 kDa) were obtained using ultrafiltration, and found that the fraction (2–5 kDa) exhibited stronger antioxidant activity than of other fractions [88]. Jahandideh et al. (2018) showed that two papain-generated antioxidant EWPPs (Tyr-Leu-Gly-Ala-Lys and Gly-Gly-Leu-Glu-Pro-Ile-Asn-Phe-Gln) were purified sequentially by ultrafiltration, gel filtration, and RP-HPLC. Adipogenic-differentiating peptides were purified sequentially from egg white proteins after ultrafiltration, C18 Sep-Pack cartridge, and cation-exchange chromatography [89]. Eckert et al. (2019) reported that the decapeptide QSLVSVPGMS (Gln-Ser-Leu-Val-Ser-Val-Pro-Gln-Met-Ser) was fractionated from EYPs by a series of purification procedures, including membrane filtration, gel filtration chromatography, and RP-HPLC, and its ACE-inhibitory and DPPH radical scavenging activities were almost two-times and over three times higher compared to those of the initial EYPs, respectively [90].

Moreover, the purified HEPs must be further identified by peptide sequencer techniques, including Edman degradation methods, mass spectrometry (MS) methods, and bioinformatics analysis. The Edman degradation method has some limitations, such as blocking N-terminal amino acids, forming a blank cycle, and being susceptible to interference [91]. MS methods are used to separate ions by their mass to charge ratio (*m*/*z*) using mass analyses, and they can also be combined with chromatography for excellent analytical accuracy and specificity [92]. The mass analyzer is the core component of MS analysis and identification, and there are commonly used mass analyzers, including quadrupole, time of flight (TOF), ion trap, and electrostatic field orbital trap (Orbitrap) [93]. Two or more mass analyzers are combined in series to enhance the speed and accuracy of the analyses. MS methods have gradually replaced the Edman degradation method as a powerful tool for peptide sequencing due to their high sensitivity and high- throughput sequencing. Currently, the most common strategies for confirming peptide sequences include matrix-assisted laser deionization time-of-flight (MALDI-TOF), liquid chromatography triple quadrupole mass spectrometry (LC-MS/MS), electrospray ionization mass spectrometry (ESI-MS), and triple quadrupole mass spectrometer (TQ-MS) [23,55,56,89,94]. Zambrowicz et al. (2015) reported that some isolated peptides, deprived from egg yolk proteins, were identified using MALDI-TOF [95]. WEKAFKDED, QAMPFRVTEQE, ERYPIL, VFKGL, AEERYP, DEDTQAMP, and RVPSL were identified and isolated from EWPs by LC-MS/MS [74,86,89]. KGGDLGLFEPTL (Lys-Gly-Gly-Asp-Leu-Phe-Glu-Pro-Thr-Leu), DEEENDQVK AEFGTEPDAKTSSSSSSSASSTATSSSSSSSSSPN (Asp-Glu-Glu-Glu-Asn-Asp-Gln-Val-Lys-Ala-Glu-Phe-Gly-Thr-Glu-Pro-Asp-Ala-Lys-Thr-Ser-Ser-Ser-Ser-Se-Ser-Ser-Pro-Asn), KPMDEEENDQV (Asn-Pro-Met-Asp-Glu-Glu-Glu-Asn-Asp-Gln-Val), and SGHLEDDSSSSSSSSVLSKIWG (Ser-Gly-His-Leu-Glu-Asp-Aspr-Ser-Ser-Ser-Ser-Se-Ser-Ser-Ser-Val-Leu-Ser-Lys-Ile-Trp-Gly) were also identified and isolated by LC-MS/MS [9,96]. Moreover, Samaraweera et al. (2014) identified ten PPPs from trypsin EYPs [56]. Additionally, a variety of databases, web resources, and software are used in bioinformatics techniques, commonly referred to as in silico analysis, to predict the bioactivity of peptides. This is accomplished by analyzing the type and quantity of amino acid residues using statistical and computational methods [97]. Currently, peptidomics and bioinformatics collaborate to provide a cost-effective and efficient approach to screening, analyzing, and predicting. UniportKB, BIOPEP, PeptideCutter, and PeptideRanker are databases dedicated to analyzing the functionality of peptide sequences [98]. Marcet et al. (2022) used LC-MS/MS to identify the EYPPs released from EYPs. Subsequently, they utilized the BIOPEP database, ExPASy ProtParam, AHTpin, and PeptideRanker to analyze the discovered EYPPs and predict their bioactivities [99]. Mohd Adam Salim et al. (2020) utilized a comprehensive integrated bioinformatics technique (PeptideCutter, PeptideRanker, and Pepsite2) to study the structure–activity link between OVAPs and ACE/DPP-4. They found eight peptides with particular activity [100]. With the development of technology, bioinformatics is gradually being utilized to study the structure–activity relationship of bioactive peptides. For peptides with well-defined structures, molecular docking and molecular dynamics simulations are commonly used to examine the biological activity mechanisms. When the peptide structure cannot be identified, the quantitative structure–activity relationship (QSAR) is employed to examine the association between the biological activity and chemical structure. Majumder et al. (2010) found that three ACE-inhibitory peptides were successfully isolated from OTFP after screening the virtual enzymatic hydrolysis products predicted by the QSAR model [101].

## 3. Biological Activities of Hen Egg-Derived Peptides

### 3.1. Antioxidant Activity

Reactive oxygen species (ROS) and free radicals are beneficial byproducts to human health. However, an excess of ROS and free radicals can cause serious oxidative damage to a cell’s structure or function, which can contribute to the onset of a number of diseases, including diabetes, aging, and neurodegenerative illnesses [102,103]. Hydrolyzed peptides from egg proteins are believed to be potent antioxidants that can either scavenge reactive oxygen species (ROS) or stop ROS from being produced [104]. Table 2 summarizes the methods used to prepare HEPs with potential antioxidant activity. HEPs may reduce oxidative damage by scavenging free radicals, blocking pro-oxidative enzymes, and chelating metal ions, among other mechanisms [10]. Currently, the most widely used methods for detecting their activity are ORAC-FL (oxygen radical absorbance capacity-fluorescein), FRAP (ferric ion-reducing antioxidant potential), ABTS (2,2′-azino-bis (3-ethylbenzothiazoline-6-sulfonic acid), and DPPH (2,2-diphenyl-1-picrylhydrazyl).

The antioxidant peptides are mainly derived from EWPPs, with a minor fraction from EYPs and other components. Dávalos et al. (2004) proposed that the antioxidant activities (ORAC-FL value of 3.8 μmol of Trolox equivalent per μmol of peptide) of EWPPs (Tyr-Ala-Glu-Glu-Arg-Tyr-Pro-Ile-Leu) might be correlated with the presence of Tyr residues in the N-terminal region [108]. AEERYP (Ala-Glu-Glu-Arg-Tyr-Pro) and DEDTQAMP (Asp-Glu-Asp-Thr-Gln-Ala-Met-Phe) identified from EWPPs also showed high ORAC values, at 3.32 ± 0.07 μmolTE/μmol and 3.14 ± 0.1 μmol TE/μmol, attributed to the presence of the termini Ala and Pro, respectively [74]. The antioxidant activity of EWPPs was linked to the presence of aromatic hydroxyls through a hydrogen atom transfer mechanism. Moreover, compared to other fractions, EWPPs with a molecular weight of 2–5 kDa exhibited a stronger antioxidant activity (DPPH radical scavenging activity, 98.93 ± 2.53%). This strong antioxidant activity may be associated with the presence of Phe, Tyr, and His, which act as proton donors when they interact with active free radicals [88]. Liu et al. (2015) also confirmed that EWPPs (Mw < 1 kDa) exhibited a significant antioxidant activity (5 mg/mL; DPPH radical scavenging activity, 69.31 ± 0.52%) [11]. As a result, the antioxidant activity of EWPPs may be primarily associated with their molecular weight and composition. The lower-weight antioxidant peptide tends to have a stronger biological effect. To further study the antioxidant mechanism under physiological conditions, Yi et al. (2017) discovered that IRW derived from OTFP could inhibit H_2_O_2_-induced cytotoxicity and ROS generation, thereby protecting Caco-2 cells from oxidative stress [109]. Another study revealed that IRW and LKP showed synergistic antioxidant effects with the phytochemical’s vitamin C, EGCG, and caffeic acid at high molar ratios [105].

EYPs and EYPPs also have excellent antioxidant activities because of their abundance of aromatic amino acids. Studies have demonstrated that the peptide sequence, molecular weights, and the presence of amino acid residues (tryptophan and tyrosine) are closely related to the radical scavenging activity [110]. Yousr et al. (2015) showed that EYGF-23, EYGF-33, and EYGF-56 were isolated and purified from EYPs, which could inhibit oxidative stress induced in a linoleic acid-oxidizing model system by radical scavenging and metal chelation [111]. Moreover, QSLVSVPGMS (Gln-Ser-Leu-Val-Ser-Val-Pro-Gly-Met-Ser) also exhibited a strong in vitro DPPH free radical scavenging activity [90]. PPPs also showed in vitro antioxidant activity by upregulating glutathione synthesis and antioxidant enzyme activity [112]. Moreover, digested PPPs still exhibited antioxidative stress properties. Shi et al. (2014) confirmed that ESMPs dramatically reduced intestinal oxidative stress in Caco-2 cells by increasing their mRNA expression [113]. And ESMPs are also considered to be a novel source of antioxidant peptides. Zhu et al. (2022) reported that ESMPPs (<3 kDa) exhibited potent antioxidant activity and found that they could protect H_2_O_2_-induced RAW264.7 cells by the modulation of the Keap1-Nrf2 pathway [114]. Therefore, low-molecular mass peptides, hydrophobic amino acids, and aromatic amino acids are essential for the antioxidant activity of HEPs.

### 3.2. Enzyme Inhibitors

Enzyme inhibitors are substances that can inhibit the activity of specific enzymes related to certain diseases in the organism, resulting in a therapeutic effect. The majority of enzyme-inhibitory sequences are derived from hen egg proteins, such as ovalbumin, ovaltransferrin, and lysozyme. Table 3 shows the HEPs with enzyme-inhibitory activities. These are angiotensin-I-converting enzyme (ACE, EC 3.4.15.1) inhibitor, dipeptidyl peptidase IV (DPP-IV, EC 3.4.14.5) inhibitor, α-glucosidase (EC 3.2.1.1) inhibitor, aminopeptidase N (APN, EC 3.4.11.2) inhibitor, xanthine oxidase (XO, EC 1.17.3.2,5) inhibitor, and tyrosinase (EC 1.14.18.1) inhibitor.

Hypertension is frequently recognized as an induction factor for CVDs, and it is deemed that ACE and oxidative stress are closely associated with hypertension [118]. HEPs exerts antihypertensive effects primarily by inhibiting ACE activity in the renin–angiotensin system (RAS). RAS consists of renin, angiotensin-converting enzyme, and angiotensinogen. ACE can accelerate the conversion of decapeptide angiotensin I into octapeptide angiotensin II, an effective vasoconstrictor [119]. ACE catalyzes the degradation of bradykinin (a vasodilating peptide) in the kallikrein–kinin system (KKS) [120]. Currently, ACE inhibition is regarded as one of the most effective treatments for hypertension. ACE-inhibitory peptides are mostly derived from ovalbumin, ovotransferrin, and lysozymes, which are often produced through controlled enzymatic hydrolysis. The ACE inhibitory peptide (IC_50_ of 3.2 μmol/L) was first identified from OVAPs in 1995 [121]. QIGLF (Gln-Ile-Gly-Leu-Phe), isolated from OVAPs, exhibited a strong ACE activity (IC_50_ of 75 µmol/L) due to its high α-helix and resistance to in vitro digestion by gastrointestinal proteases [84]. Therefore, the secondary structure of HEPs also contributes to their ACE-inhibitory activity. The antihypertensive effects of the peptide QIGLF are associated with aldosterone-regulated sodium reabsorption, mitophagy, gap junction, and tight junction. Memarpoor-Yazdi et al. (2012) prepared LMPs with ACE-inhibitory activity by gastrointestinal tract proteases and further identified dodecapeptide (FESNFNTQATNR Phe-Glu-Ser-Asn-Thr-Gln-Ala-Thr-Asn-Arg), with an IC_50_ value of 0.03 mg/mL [40] However, Rao et al. (2012) reported that two ACE-inhibitory peptides (Met-Lys-Arg and Val-Ala-Trp, with IC_50_ values of 25.7 ± 0.2 and 2.86 ± 0.08 μM, respectively) were obtained from LMPs [115]. Among these, the positively charged amino acids of Met-Lys-Arg could also promote peptides binding to ACE [115]. The impact of explicit ACE-inhibitory sequences was more pronounced compared to that of hydrolysates, and short-chain peptides may exhibit a superior performance compared to long-chain ones. Furthermore, tripeptides with proline or aromatic residues (Trp/Phe/Tyr) at the C-terminus and branched-chain amino acids (Val/Ile/Leu) exhibited an efficient ACE-inhibitory action [122]. RVPSL was obtained from EWPs and showed an outstanding ACE-inhibitory activity, attributed to the presence of Leu at the C-terminus [122]. Yu et al. (2020) further demonstrated that ACE-inhibitory peptides synthesized chemically from the hydrolysate of EWPs exhibited a potential antihypertensive effect in spontaneously hypertensive rats (SHRs) [123]. And Aleksandra et al. (2014) reported that EYPs exerted a strong ACE inhibitory activity (IC_50_ of 9.4 µg/mL), which has been attributed mainly to the glutamic acid residue at the C-terminal positions [95]. Moreover, it has been found that oxidative stress is related to vascular damage in hypertensive patients, due to the degradation of excessive reactive oxygen species (ROS) and nitric oxide (NO) [124]. Zhang et al. (2020) reported that LAPYK, identified from EWPPs, had an ABTS scavenging IC_50_ of 5.29 ± 0.24 μM and ORAC of 1.50 ± 0.04 μM TE/μM, and found that LAPYK has potential to exhibit antihypertensive activity [118]. Another study confirmed that the egg white-derived peptide QIGLF could decrease the systolic blood pressure of hypertensive rats (SHRs), which may be involved in the inhibition of Na reabsorption and oxidative stress [84].

Currently, DPP-IV inhibitors (DPP-IVi) are effective treatment options for patients with type 2 diabetes mellitus. DPP-IV mainly regulates blood glucose levels by inhibiting the degradation of glucagon-like peptide-1 (GLP-1) and glucose-dependent insulinotropic polypeptide (GIP) [125]. Zhao et al. (2020) isolated the DPP-Ivi peptides ADF (Ala-Asp-Phe), MIR (Met-Gly-Arg), and FGR (Phe-Gly-Arg) from hen eggs. These peptides exhibited IC50 values of 16.83 mM, 4.86 mM, and 46.22 mM, respectively, maybe due to hydrogen bond interactions [85]. The suppression might also be attributed to the presence of the C-terminals Arg and Phe [126]. Wang et al. (2019) confirmed that the three peptides IRDLLER (Ile-Arg-Asp-Leu-Leu-Glu-Arg), YAEERYP (Try-Ala-Glu-Glu-Arg-Tyr-Pro), and IRNVLQPS (Ile-Arg-Asn-Val-Leu-Gln-Pro-Ser) identified from EWPPs were demonstrated as DPP-IV inhibitors (IC_50_ values of 186.23 ± 15.25, 340.62 ± 4.73, and 598.28 ± 15.12 μmol/L, respectively), which were mainly attributed to form hydrogen bonds, charge interactions, and π–π interactions with DPP-IV [116]. DPP-IV activity was detected in ADF, MIR, and FGR that were isolated from hen egg proteins (16.83 mM, 4.86 mM, and 46.22 mM, respectively). This activity may have been caused by hydrogen bond interactions.

Furthermore, α-glucosidase is the crucial enzyme for the final step of dietary carbohydrate digestion and glucose release [127]. α-glucosidase inhibitors can reduce glucose absorption to regulate blood sugar. Nowadays, some α-glucosidase inhibitors have been identified from egg white proteins. Yu et al. (2011) reported that RVPSLM (Arg-Val-Pro-Ser-Leu-Met, IC_50_ of 23.07 μmol/L), a peptide, inhibited α-glucosidase derived from egg white ovomucin pepsin hydrolysates through a combination of hydrogen bond and hydrophobic interactions [83].

Additionally, the aminopeptidase N (APN)-inhibitory peptides CNR (Cys-Asn-Arg), CDR (Cys-Asp-Arg), and GEF (Cys-Glu-Phe), with IC_50_ values of 8.94, 6.42, and 61.7 mM, respectively, were isolated and synthesized from egg white hydrolysates prepared by proteases and pepsin [117]. This suppression was suggested to have been caused by contact between inhibitory peptides and APN residues (Arg363, Arg381, Gly352, and Ala353).

Yu et al. (2022) reported that XO-inhibitory peptides, identified from egg white protein hydrolysates, had a favorable implication in the treatment of some diseases, such as cancer, inflammation, and tumors. And hydrogen bond and attractive charge interactions promoted to peptides bound to the active center [87].

Yap et al. (2020) reported that the monophenolase and diphenolase activity inhibition values of EWPPs were 45.9% and 48.1%, respectively, at the optimized conditions (55% trypsin, 45% chymotrypsin, S/E 10:1 *w*/*w*, 2 h; 100% trypsin, S/E 22.13:1 *w*/*w* and 3.18 h), and EWPPs were able to bind to H85, H94, H259, H263, and H296 (hotspots for active residues) as well as F92, M280, and F292 (stabilizing residues) of tyrosinase based on the structure–activity relationship analysis [128]. PPPs prepared with HTMP-T-S (HTMP pretreatment-trypsin-sterilization hydrolysis) showed the most potent inhibition of tyrosinase and elastase, with the inhibition rates of 88.09% and 70.67% in sequence, respectively, which reduced the melanin content in B16F10 melanoma cells [82].

### 3.3. Antibacterial Activity

Lysozyme, ovomucin, ovotransferrin, and vitelline phosphoprotein are antibacterial proteins found in eggs that are widely employed as natural antimicrobials against a variety of microorganisms. Currently, many studies have reported the antimicrobial activity of HEPs (Table 4), which are released after the partial hydrolysis of exogenous proteases. Antimicrobial mechanisms largely involve the interaction of positively charged amino groups with the negatively charged microbial cell membrane, resulting in membrane rupture and intracellular material [129]. Two antimicrobial peptides purified from LMPs, Ile-Val-Ser-Asp-Gly-Asp-Gly-Met-Asn-Ala-Trp, inhibited the Gram-negative bacterium *Escherichia coli* K-12 by damaging the membrane via the C-terminal helix, and His-Gly-Leu-Asp-Asn-Tyr-Arg inhibited *Staphylococcus aureus* 23–394 by the N-terminal helix and loop [130]. Shen et al. (2020) proposed that KSWKKHVVSGFFLR (Lys-Ser-Trp-Lys-Lys-His-Val-Val-Ser-Gly-Phe-Phe-Leu-Arg) and KSWKKHVVSGFFLRLWVHKK (Lys-Ser-Trp-Lys-Lys-His-Val-Val-Ser-Gly-Phe-Phe-Leu-Arg-Leu-Trp-Val-His-Lys-Lys) identified from LMPs could also destroy the cell membranes’ integrity by membrane depolarization and increasing membrane permeability, and P3R1 (MIC, 125 μg/mL) interacted with the DNA of the enterica serotype Typhimurium, resulting in DNA aggregation [131]. Kobayashi et al. (2004) reported that the ovomucin glycopeptide (OGP) had a specific binding site for *Escherichia coli* O157: H7 composed of sialic acid, which can bind to *Escherichia coli* to achieve the purpose of bacteriostasis [132]. Moreover, it has been reported that an antimicrobial domain is a characterized structure of the transferrin family [133]. AGLAPYKLKPIA isolated from OTFP exhibited antimicrobial activity against various Gram-positive and Gram-negative pathogenic bacteria (*Escherichia coli* and *Staphylococcus aureus*) by altering membrane permeability and disrupting cell surface morphology [134]. Memarpoor-Yazdi et al. (2012) reported that NTDGSTDYGILQINSR (Asn-Thr-Asp-Gly-Ser-Thr-Asp-Tyr-Gly-Ile-Leu-Gln-Ile-Asn-Ser-Arg identified from LMPs) was toxic to *Escherichia coli* and *Leuconostoc mesenteroides* bacteria, displaying a minimal inhibitory concentration (MIC) of around 355.64 ± 2.2 and 442.25 ± 2.8 μg/mL, respectively [40]. Pimchan et al. (2023) found that KGGDLGLFEPTL (Lys-Gly-Gly-Asp-Leu-Gly-Leu-Phe-Glu-Pro-Thr-Leu from EYPs had high antibacterial activity against *Staphylococcus aureus* at an MIC of 2 mmol/L [9]. Choi et al. (2004) reported that PPPs effectively chelated metal ions outside the *Escherichia coli* cell membrane, triggering apoptosis and cell death [135].

### 3.4. Other Activities

Egg-derived peptides have anti-inflammatory, immunomodulatory, and anti-cancer activities, as well as chelated minerals. Currently, studies have shown that pro-inflammatory factors could alter the intestinal mucosal barrier function by some pathways, including NF-κB, mitogen-activated protein kinase (mitogen-activated) protein kinase (MAPK), Janus kinase-signal transduction and transcription activator (JAK-STAT), and others [136]. Kim et al. (2020) reported that the expression of inducible nitric oxide synthase (iNOS) mRNA and the production of iNOS (62.35% at 2 mg/mL) were both inhibited by OVAPs in LP-stimulated RAW 264.7 cells [137]. Sun et al. (2016) investigated the anti-inflammatory activity of various OVAPs and their components in human skin fibroblasts. They found that desalinated alkaline protease hydrolysates could significantly reduce the expression of TNF-induced ICAM-1 [138]. Majumder et al. (2013) proposed that transferring-derived peptides (IRW IIe-Arg-Trp) and IQW IIe-Gln-Trp) could effectively relieve the inflammatory response and oxidative stress induced by tumor necrosis factor (TNF) [139]. IRW and IQW significantly inhibited the upregulation of the TNF ICAM-1. IRW also suppressed the upregulation of the vascular cell adhesion molecule-I (VCAM-1), thus inhibiting the NF-κB pathway and showing anti-inflammatory activity. Moreover, some EYPs can also exhibit anti-inflammatory activity via the inhibition of the production of nitric oxide (NO), pro-inflammatory cytokines (tumor necrosis factor-α, TNF-α; interleukin-1β, IL-1β; and interleukin-6, IL-6), and iNOS [140]. PPPs might inhibit the NF-κB signaling pathway in intestinal epithelial cells and the macrophage cell line RAW 264.7 by regulating pro-inflammatory factors (IL-8, IL-6, MCP-1, IL-12, TNF-α, and IL-1β) [141]. Lee et al. (2022) also confirmed that PPPs enhanced the mRNA expression of the pro-inflammatory cytokines IL-6 and TNF-α. They exhibited anti-inflammatory activity by activating anti-Toll-like receptor-mediated MAPK signaling in RAW 264.7 cells [142]. Additionally, Rzhepakovsky et al. (2021) proposed that HEPs could have an adjuvant anti-inflammatory effect against arthritis in rats by the inhibition of protein denaturation, an effect on membrane stabilization, and proteinase inhibitory activity, and found that HEPs had better recovery effects on inflammation compared to the anti-inflammatory drug diclofenac sodium (5 mg/kg) [143].

In addition, some egg-derived components may stimulate the secretion of TNF-α release to further enhance the phagocytic activity of macrophages. By using phagocytosis to kill a pathogen directly, macrophages can also attract other immune cells by releasing cytokines, which are essential components of the immune system. The immunomodulatory properties of bioactive peptides derived from ovotransferrin have also been demonstrated. Moreover, they could reduce the expression of costimulatory molecules (MHC-II, CD83, and CD86) and IL-12p70 and TNF-α production, thus inhibiting the LPS-induced phenotypic and the functional maturation of murine bone marrow-derived dendritic cells [144]. Kazana et al. (2022) proposed that yolkin, a peptide complex discovered from egg yolk, could inhibit the proliferation of mouse bone marrow-derived macrophages and upregulate the expression of CD80/CD86 based on MAP and phosphoinositide 3-kinase/protein kinase B (PI3K/Akt) activation [145]. OVAPs activated resting macrophages via the MAPK signaling pathway, boosting the immune response [12]. Lee et al. (2022) reported that EYPs could increase the production of NO TNF-α and IL-6 in macrophages, which in turn increased the phagocytic activity [146]. Yolkin also stimulated whole blood cells to release TNF-α, IL-6, IL-10, and IL-1β for immunoregulation [147,148]. Additionally, OTFPs prepared with promo 278P and thermolysin displayed a potent cytotoxic activity against HT-29, LoVo, and HeLa cell lines [77].

Yi et al. (2003) also reported that EWPPs could inhibit P3881D cell proliferation by arresting the cell cycle at the G2/M phase to achieve the effects of anti-cancer [149]. Phosphopeptides isolated from the egg yolk demonstrated efficient chelating abilities with metal ions due to their richness in phosphorylated amino acid residues. Among others, there are numerous studies on peptide–calcium chelates or peptide–iron chelates. Liu et al. (2023) reported that an iron-chelating peptide prepared from egg yolk hydrolysates exhibited high iron-chelating activity (87.32%) [150]. Phosphorylated serine, glutamate, amino, and carboxyl groups in the peptide chain might play a vital role in the formation of peptide–iron chelates. High calcium-chelating PPPs (DEEENDQVK Asp-Gu-Gu-Gu-Asn-Asp-Gln-Val-Lys) were synthesized using amino acid sequences from PV hydrolysates, with a calcium-binding capacity of 151.10 ± 3.57 mg/g [67]. It was hypothesized that the carboxyl oxygen and amino nitrogen atoms were binding sites in the peptide. PPP–calcium was reported to improve calcium absorption in the gut and bone deposition [81]. Moreover, PPPs can also accelerate bone mineralization. Zhao et al. (2020) proposed that PPPs, obtained from a HTMP pretreatment and trypsin hydrolysis combination, promoted the proliferation and differentiation of MC3T3-E1 cells by increasing the expression of OPG/RANKL signaling pathway-related genes [151]. Other activities of HEPs discussed in this review are summarized in Table 5.

## 4. Potential Applications of Peptides

HEPs have exhibited enormous potential as bioactive components. There is increasing interest in the commercial application and research of HEPs. For instance, López-Martínez et al. (2021) proposed that a novel ice-cream was obtained using EWPPs, which can be used as a healthy alternative to commercial dairy ice-cream [156]. EWPPs also increase the foaming and stability of sterilized liquid eggs and enhance their utilization in the bakery food industry [157]. Beyond that, HEPs with antioxidant activity also have development potential as substitutes for synthesizing antioxidants and multifunctional raw materials for producing healthcare products, including functional foods, cosmetics, and drugs. HEPs have antibacterial activity, such as excellent performance against *E. coli*, and exhibit a great potential in the development and application of veterinary drugs, antibacterial agents, and anti-inflammatory functional foods. Furthermore, two clinical double-blind trials demonstrated that ESMPs can improve skin wrinkles in women (30~60 years old) and be used as a dietary supplement to relieve the symptoms of osteoarthritis patients [158,159]. And some HEPs can enhance the absorption of dietary iron and calcium. Sun et al. (2017) analyzed a calcium-binding peptide (DHTKE) from HEPs, and the calcium absorption of the DHTKE–calcium complex was seven times higher than the free-calcium control in Caco-2 cells [68]. HEPs can be used as a biopreservative. LM is the most stable anti-infection agent in egg whites, and the antibacterial activity of lysozymes might be further enhanced by exposing the antibacterial portions of the protein independent of its catalytic function [22].

Moreover, highly soluble halophilic His-rich metal-binding proteins were combined with an insolubilized Nona-peptide derived from hen egg white lysozymes, and they could be used for the efficient biosorption and recovery of environmental metal ions, serving as a new cost-effective metal-ion adsorbent [160]. Therefore, HEPs have several applications in both the food and non-food sectors.

### 4.1. Intervention in Intestinal Health

Due to the influence of the amino acid content and structural properties, there might be differences in the bioactivities and bioavailability of peptides between in vitro and in vivo conditions. Therefore, it is important that HEPs cross the gastrointestinal (GI) barrier intact and pass through the intestinal membrane into the bloodstream to reach the site of action [161]. Several studies have suggested that some HEPs have a good resistance to digestive electrolysis. For example, the antihypertensive HEPs TNGIIR (Thr-ASN-Gly-IIe-Arg), RVPSL (Arg-Val-Pro-Ser-Leu), and QIGLF (Gln-IIe-Gly-Leu-Phe)) could hardly be hydrolyzed by gastrointestinal enzymes, and it was found that the paracellular pathway of TJs was the main transport route for TNGIIR across Caco-2 cell monolayers [162]. Some HEPs are further hydrolyzed into short peptides during gastrointestinal digestion. These peptides exert their intended biological action upon absorption into the systemic circulation. Two peptides isolated from β-lactoglobulin hydrolysates (LLF and LQKW) showed ACE-inhibitory activity. LL (Leu-Leu) and LQK (Leu-Gln-Lys), obtained after gastrointestinal digestion, retained potent in vitro ACE-inhibitory and increased antihypertensive activity [163]. Ovalbumin-derived ACE-inhibitory peptides decreased in activity and showed antihypertensive activity after gastrointestinal digestion [164]. Moreover, the inhibition of ACE might not be the unique regulatory mechanism of antihypertensive HEPs [165]. For instance, renin inhibition has been identified as an effective approach to block RAAS (renin-angiotensin-aldosterone system) [166]. Renin can catalyze the hydrolysis of angiotensinogen to produce angiotensin I-10 (Ang I) in the RAAS; then, angiotensin II (Ang II) is obtained with the action of ACE and Ang I-10, leading to blood vessel contractions [167]. Yu et al. (2014) proposed that a pentapeptide (RVPSL) from egg proteins decreased renin mRNA expression in the kidneys of spontaneously hypertensive rats to regulate blood pressure [168]. And angiotensin-converting enzyme 2 (ACE2, EC 3.4.17.23) plays beneficial roles in the renin angiotensin aldosterone system, and it can also convert Ang I-10 into Angiotensin 1–9, reducing the interaction between Ang I-10 and ACE [169,170]. Liao et al. (2018) confirmed that IRW derived from EWPs exhibited antihypertensive activity via upregulating the ACE2 content in SHR [171]. The gut is the primary organ for the digestion and absorption of nutrients. Recently, intestinal health issues have received significant attention. Studies indicate that an imbalance in the intestinal barrier or intestinal damage can lead to intestinal diseases, such as ulcerative colitis and Crohn’s disease [172]. An intact intestinal barrier and healthy gut microbiota are essential indicators of gut health [173]

**Figure 1:**
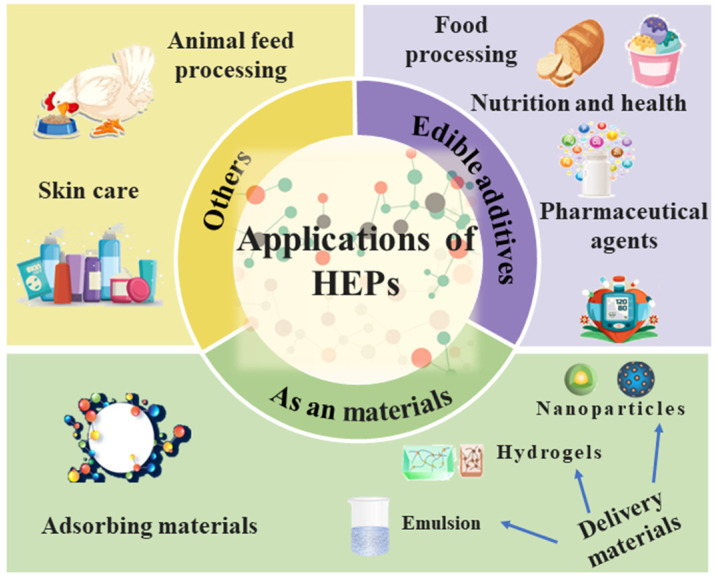
Application of HEPs in the food and non-food sectors.

Food-derived peptides have been demonstrated to be important in maintaining intestinal health. Soybean peptides could be used as beneficial modulators of gut microbiota, altering the microbiota composition by increasing probiotic bacteria and decreasing Bacteroides [174]. Milk-derived peptides can regulate immune cytokine production, anti-inflammatory, and humoral immune activities. The NF- κ B inhibitory peptide obtained from β-casein and whey protein hydrolysates both inhibited small bowel neoplasms [175]. Furthermore, egg-derived peptides were found to modulate intestinal health. The oral dose of ESMPs that could alleviate the severity of clinical symptoms was significantly reduced. OVMPs can stimulate the development of B. infantis and increase lactate production [176]. Fermented egg–milk beverages can improve colitis symptoms in mice by increasing body weight, goblet cells, and crypts [177]. In another study, obese rats were fed EWPPs in nutritional interventions, and it was found that the faucal concentration of lactic acid, oxidative stress, and inflammation markers in obese Zucker rats reduced [178]. In summary, HEPs enhance the function of the intestinal barrier function and regulate the composition of the gut microbiota by controlling the secretion of pro-inflammatory factors and mucins, thus preventing enteric diseases. Thus, HEPs are expected to provide novel strategies to maintain gut health and prevent associated diseases. However, most studies are still limited in vitro, and how HEPs modulate the gut microbiota in vivo and impact the metabolism of microorganisms are not fully understood. Furthermore, there are possible substitution or synergistic effects between HEPs and endogenous proteins in maintaining the intestinal mucosal barrier (see Figure 1).

### 4.2. Encapsulant Materials for the Delivery of Bioactives

Moreover, food-derived peptides have gained popularity in the field of functional factors and drug delivery because of biocompatibility and biodegradability. HEPs are a natural mixture that is resistant to the action of digestive enzymes because they contain various binding sites and functional groups. They can self-assemble or co-assemble with other polymers to form specific polymers, which shows a synergistic effect in health supplements [179,180]. Polymers exist in the form of solutions, emulsions, self-assembled nanoparticles, or films [181]. It was reported that π-π stacking, hydrogen bonding, and hydrophobic/hydrophilic interactions play significant roles in the self-assembly of peptides [182]. HEPs can also be used as materials to form a delivery system. Self-assembling nanoparticles composed of EYPs were widely used as an emulsifier to prepare O/W emulsions due to their good surface activity [183]. Curcumin-loaded nanocomposites were co-assembled by egg white-derived peptides and a polysaccharide–protein complex, showing antioxidant activity, and it was found that the presence of EWPPs (Cur-EWPP NPs, 26.26%) significantly enhanced the cellular antioxidant activity compared to free Cur (2.26%) [184]. Liu et al. (2021) proposed that high-density lipoprotein peptides could be a new self-assemble nanoemulsifier. They tended to act as particulate emulsifiers (pH values of 3, 5, and 7) and polymeric emulsifiers (at an alkaline pH) [183]. Du et al. (2019) discovered a natural edible nano-Pickering emulsifier made from self-assembled egg yolk peptides (EYPNs) that can create stable and controllable food Pickering nanoemulsions (dynamic light scattering diameter < 200 nm and polydispersity index < 0.2) for use in foods, pharmaceuticals, and cosmetics [185]. And EYPNs could act as an efficient particulate stabilizer for the development of food-grade aqueous foams, which were a highly pH-responsive system that were highly stable at a pH of 6.0–9.0 but unstable at low pH values (2.0–5.0) [186]. Some egg white-derived peptides were combined with carboxymethyl chitosan to form excellent colloid properties and increase the water solubility of a curcumin-loaded delivery system co-assembled by HEPs, carboxymethyl chitosan, and γ-cyclodextrin, which was mainly driven by the complicated hydrogen-bonding network and hydrophobic interaction [187]. HEPs can be used to synthesize nanoparticles, which were applied to encapsulate calcium or zinc to prevent ion precipitation and, therefore, facilitate the absorption of this component [188,189].

Furthermore, self-assembled peptides showed a better enzymatic degradation and in vitro biocompatibility than monomeric units, broadening their applications in vitro. Self-assembled peptide materials outperform alternative carrier materials in terms of biocompatibility and structural flexibility. Egg-derived peptides are in high demand due to their safety, hydrolysis responses, efficacy, low cost, and ease of preparation [190]. Hence, egg-derived peptides have a great potential in the encapsulation of food and pharmaceutical applications. However, the assembly or self-assembly of egg-derived peptides is still in its infancy and has some limitations, including difficulties in verifying their specific peptide fragments and sequence characteristics after hydrolyzation. Therefore, further studies are necessary to address these issues.

## 5. Conclusions

This review focuses on the preparation of HEPs, their biological activities, and potential applications. According to research, the most prevalent method for producing HEPs is enzymatic hydrolysis, and genetic engineering is expected to become a major trend in HEPs manufacture. Afterward, we explore the structure-activity relationship of HEPs to reveal various functional properties. Furthermore, in vivo and in vitro studies have revealed that the biological activities of HEPs are closely related to their amino acid sequence and molecular weight, laying the groundwork for future research into the molecular mechanisms of HEPs. The above-mentioned facts are mainly derived from in vitro and animal investigations, while clinical evidence is sparse. Further clinical trials are required to thoroughly examine the efficacy and bioavailability of HEPs in vivo. Moreover, HEPs have great potential in various fields, including food, drugs, and biological materials. It is of great value for maintaining human nutrition and promoting human health.

## Figures and Tables

**Table 1:** Preparation methods of HEPs.

Methods	Protein	Preparation	Evaluation Indicator	Results	References
Enzymatic hydrolysis	Eggshell membrane protein	Alcalase: E/S 1% *w*/*w* at pH 10, 55 °C for 4 h. Proteases S: E/S 2% *w*/*w*, pH 10 at 55 °C for 12 h. Alcalase + Protease S	Total nitrogen recovery DPPH radical scavenging activity Hydroxyl radical scavenging activity	65.60 ± 0.43% 42.74% 25.5%	[43]
		Alcalase from *Bacillus licheniformis:* E/S (*v*/*v*) 2.2%, pH 7.6, 55 °C, 6 h. Viscozyme L: E/S (*v*/*v*) 1.90, pH 4.6, 50 °C, 6.61 h. Protease from *Bacillus amyloliquefaciens:* E/S (*v*/*v*) 5.3%, pH 6.6 h, 50 °C, 2.90.	ACE inhibitory activity	IC_50_ (μg/mL: 43.0 ± 8.5 63.0 ± 4.2 43.0 ± 8.5	[72]
		Recombinant LasB_ME4 (*Pseudomonasaeruginosa*) E/S 3, pH 6.5, 50 °C for 24 h, with shaking at 140 rpm.	Soluble peptides and proteins (mg/mL)	0.502 ± 0.016	[73]
	Egg white protein	Papain: pH 6.0, 50 °C, 5 h.	DPPH scavenging activity The recovery of the 3 h papain hydrolysate	73.14% 50.62%	[11]
		Protease P: E/S (*w*/*w*) 25:1, pH 7.5, 45 °C, 3 h.	DH ORAC (μmol TE/mg) ABTS (μmol TE/mg)	93.3% 1.28 ± 0.06 1.61 ± 0.00	[74]
		Ultrasound-assisted alkali treatment: pH 8.5, 55 °C, 20 min. Alcalase: E/S 5% (*w*/*v*), pH 8.5, 55 °C, 5 h.	Foaming ability Foam stability Oil absorption capacity Water absorption capacity	Compared to unpretreated enzymolysis, these properties increased by 88.5%, 228.7%, 102.6%, and 67.4%, respectively	[75]
	Ovotransferrin	Promod 278P: E/S (*w*/*w*) 1:50, 45 °C, 3 h.	ACE-inhibitory activity Cytotoxic activities	76.82 ± 1.28% IC_50_ (mg/mL) MCF-7 cells: 10.05 ± 1.55 HeLa cells: 3.45 ± 0.94 HepG2 cells: 4.43 ± 1.87 HT-29 cells: 4.92 ± 0.63 oVo cells: 10.43 ± 3.91	[76]
		Sonication pretreatment (60 Hz for 30 s) Thermolysin: 5% (*w*/*v*), pH 8, 60 °C, 3 h. Thermolysin with sonication	ORAC (μmol/mg)	1.95 ± 0.02	[59]
		Promod 278P + Thermolysin: E/S 1:50 (*w*/*w*), 6 h.	Cytotoxic activity	IC_50_ (mg/mL) AGS, LoVo, HT-29, and HeLa: 0.79, 0.78, 0.92, and 0.78, respectively	[77]
		Papain: E/S (*w*/*w*) 5%, pH 7.0, 50 °C for 6 h.	Biofilm eradication activities	Compared to the control, the treatment of OTFP (500 μg/mL) reduced the biofilm formation rates by 13.76%, 19.18%, and 39.80% and inhibited the metabolic activities by 27.84%, 57.85%, and 65.71% in *L. monocytogenes* ATCC 15313, H7962, and NADC Scott A, respectively	[78]
		Elastase: E/S 1% (*w*/*v*), pH 7.8, 25 °C, 24 h. α-chymotrypsin: E/S 1% (*w*/*v*), pH 6.5, 37 °C, 3 h.	Fe^3+^-chelating activities	1.06 ± 0.88% 1.25 ± 0.24%	[79]
	Lysozyme	Alcalase: E/S (*w*/*w*) 0.5%, pH 7.0, 37 °C, 6 h.	Oxygen radical absorbance capacity-fluorescein (ORAC-FL) assay DPPH radical scavenging activity	IC_50_ 0.463 μmol TE/moL 1.698 μmol TE/mg	[80]
		Trypsin + Papain E/S (*w*/*w*) 1:20, pH 7.5, 37 °C, 2 h.	DPPH radical scavenging activity Radical scavenging assay (μmol (TE)/mg)	Trypsin + Papain 64.2 ± 2.95% 2.82 ±0.14 μmol TE/mg	[40]
	Phosvitin	Enzymatic treatments: Phosphatase: pH 4.8, 37 °C for 15 h. Pancreatin: pH 7.5. Pepsin A: pH 2.3.	The degree of dephosphorylation Angiotensin-converting enzyme (ACE)-inhibitory antioxidant activity	63% Dephosphorylated phosvitin: increased by 52% Protease-treated phosvitin: increased by 39%	[54]
		Alkaline treatment: 0.1 mol/L NaOH, at 37 °C for 4 h. Trypsin: E/S (*w*/*w*) 1:50, pH 8.0, 37 °C, 24 h.	Amino acid peptides Phosphate retention of phosvitin Phosphopeptides	10~20 35%	[52]
		Trypsin: pH 8.0, 40 °C, 6 h. Multifect 14 L: pH 8.0, 70 °C, 6 h. Trypsin + Multifect 14 L: The substrate/enzyme ratio was 50:1.	DH Number of peptides	26.01 ± 1.23% 164	[55]
		High-temperature and mild pressure (HTMP): 121 °C at 0.1 MPa, 30 min. Trypsin: pH 8.0, 37 °C. Thermolysin: pH 8.0, 68 °C. Trypsin + thermolysin: The same enzyme: E/S (1:50, m/m) and incubation time (8 h).	DH The calcium-binding rate of phosvitin Phosphopeptides	45.55% 43.01%	[81]
		HTMP: 121 °C and 1.5 atm for 60 min. Trypsin: pH 8.0, 37 °C. Trypsin—sterilization hydrolysis: The same enzyme: E/S (1:50, m/m) and incubation time (6 h)-	Effects of phosvitin phosphopeptides on the melanin synthesis in α-MSH-stimulated-B16F10 cells Effects of phosvitin phosphopeptides on the elastase activity Effects of phosvitin phosphopeptides on the NO production in LPS-stimulated RAW 264.7 cells	HTMP-T-S at the concentration of 3 mg/mL suppressed the melanin content by 38.58% HTMP-T-S at a concentration of 50 mg/mL inhibited the elastase activities by 70.67% HTMP-T-S at the concentration of 5 mg/mL reduced NO production by 76.69%	[82]
Microbial fermentation	Eggshell membrane protein	*Lactobacillus plantarum*: E/S (*W*/*V*) 5%, 30 °C at 120 rpm initial pH 8.0, 30 °C, 30 h.	DH Foaming capacity Emulsification activity	25.1% 36.7% 94.6 m^2^/g	[65]
	Egg white protein	Lactobacillus sp E/S (*v*/*v*) 1:1, 37 °C. Addition of different-level milk powder 2% and fermentation times, 24 h.	Antioxidant activity	3.92 ± 0.56%	[63]
	Phosvitin	*Streptococcus thermophiles* to *Lactobacillus bulgaricus* was 1:1. E/S (*w*/*w*) 1%, pH 6.7, 42 °C, 6 and 9 h.	Surface hydrophobicity Emulsifying properties The consumption of NaOH	Fermentation time: 6 h 2.04 60.49 min Fermentation time: 9 h 0.90 mL	[64]
Chemical synthesis	Egg white protein	AAPPTEC 396 Automated Peptide Synthesizer. The 9-Fluorenylmethoxycarbonyloxy (Fmoc)-protected amino acid synthesis method peptide (RVPSLM).	α-glucosidase inhibitory activity	IC_50_ 23.07 μmol/L	[83]
		Fmoc-protected amino acid synthesis method. AAPPTEC: Apex 396 automated peptide synthesizer. Peptide QIGLF.	ACE-inhibitory activity	IC_50_ 75 µmol/L	[84]
		Fmoc solid-phase peptide conditions. AAPPTEC: Apex 396 peptide synthesizer. Peptide MIR.	Dipeptidyl peptidase IV-inhibitory activity	IC_50_ 24.97 ± 0.80 mmol/L	[85]
	Ovotransferrin	Solid.phase peptide procedure. FMOC-protected amino acids synthesis method. Peptide RVPSL (328–333).	ACE-inhibitory activity	IC_50_ 20 μmol/L	[86]
	Ovalbumin	AAPPTEC: Apex 396 peptide synthesizer. Peptide EEK.	XO-inhibitory activity	IC_50_ 141 μmol/L	[87]
	Lysozyme	AEERYP	ORAC (μmolTE/mg)	IC_50_ 4.35 ± 0.09	[74]
	Phosvitin	Solid-phase peptide synthesis method. Peptide DEEENDQVK.	Calcium-binding capacity	151.1 mg/g	[67]

**Table 2:** Antioxidant peptides identified from hen egg proteins.

Protein	Peptide Sequence	Test Method	Experimental Results	References
Ovalbumin	AEERYP DEDTQAMP	Oxygen radical absorbance capacity	4.35 ± 0.09 μmolTE/μmol 3.47 ± 0.12 μmolTE/μmol	[74]
	YAEERYPIL SALAM	The mouse macrophage cell line RAW 264.7: radical-scavenging activity	3.81 µmol Trolox/mg protein 2.66 µmol Trolox/mg protein	[18]
	FFGFN MPDAHL	DPPH radical scavenging activity	IC_50_ 80 mmol/L 60 mmol/L	[11]
Ovotransferrin	WNIP GWNI	Oxygen radical absorbance capacity	15.47 ± 0.68 μmolTE/μml 13.90 ± 1.05 μmolTE/μmol	[59]
	IRW	Oxygen radical absorbance capacity	6.63 ± 0.37 μmol TE/μmol	[105]
Lysozyme	WRNRCKGTD AWIRGCRL WIRGCRL VAWRNRCKGTD	Zebrafish larvae: oxygen radical absorbance capacity-fluorescein assay	IC_50_ 3123 µmol TE/µmol 2743 µmol TE/µmol 2393 µmol TE/µmol 1970 µmol TE/µmol	[106]
Apovitellenin-1	YINQMPQKSRE	DPPH radical scavenging activity Ferric-reducing antioxidant power	2.3 µmol Trolox_eq_/mg protein 37.4 µg Fe^2+^/mg	[95]
Egg yolk protein	LAPSLPGKPKPD	Free radical scavenging activity Ferric-reducing antioxidant power	6.03 µmol Trolox_eq_/mg protein 296.07 μg Fe^2+^/mg protein	[107]

**Table 3:** Enzyme-inhibitory of HEPs.

Enzyme-Inhibitory Activity		Protein	Peptide Sequence	Test Method	Experimental Results	References
Angiotensin-converting enzyme (ACE)-inhibitory activity		Myosin Myosin Ovalbumin	ADF MIR FGR	ACE-inhibitory activity assay	IC_50_ 27.75 ± 0.90 mmol/L 24.97 ± 0.80 mmol/L 66.98 ± 1.40 μmol/L	[85]
		Ovotransferrin	RVPSL	ACE activity assay	IC_50_ 20 μmol/L	[86]
		Lysozyme	MKR RGY VAY	Stability of the synthetic peptide-inhibiting ACE	IC_50_ 25.7 ± 0.2 μmol/L 61.9 ± 0.1 μmol/L 2.86 ± 0.08 μmol/L	[115]
Dipeptidyl peptidase IV (DPP-IV)-inhibitory activity		Myosin Myosin Ovalbumin Lysozyme	MIR ADF CDR FGR	DPP-IV-inhibitory assay	IC _50_ 4.86 mmol/L 16.83 mmol/L 24.49 mmol/L 46.22 mmol/L	[85]
		Egg white protein	IRDLLER YAEERYP IRNVLQPS	DPP-IV-inhibitory activity assay	186.23 ±15.25μmol/L 340.62 ± 4.73 μmol/L 598.28 ± 15.12 μmol/L	[116]
α-glucosidase-inhibitory activity		Ovotransferrin	RVPSLM TPSPR	α-glucosidase-inhibitory activity	IC _50_ 23.07 μmol/L 40.02 μmol/L	[83]
Aminopeptidase N (APN)-inhibitory activity		Ovotransferrin Ovomucoid Avidin	CDR CNR GEF	APN-inhibitory assay	IC _50_ 8.94 mmol/L 6.42 mmol/L 61.7 mmol/L	[117]
Xanthine oxidase (XO)-inhibitory activity		Ovalbumin	EEK EEAK WPPKN ADIYTE	XO-inhibitory activity assay	IC_50_ 0.4 mg/mL 0.58 mg/mL 17.75 mg/mL 19.01 mg/mL	[87]
Tyrosinase-inhibitory activity	Monophenolase inhibitory activity	Ovalbumin Ovalbumin Ovalbumin-related protein Y	ADHPF AFKDEDTKAMPF ILELPFASGDLLML	The *p*-values of mushroom tyrosinase-peptide-binding interactions A smaller *p*-value signifies a higher potential of the peptide binding to the enzyme	0.002658 0.02053 0.03464	[82]
	Diphenolase activity	Ovotransferrin Ovomucin Ovomucin	SDFHLFGPPGK FDGRSR FNCSSAGPGAIGSEC		0.009412 0.01312 0.01614	

**Table 4:** Antimicrobial activity of HEPs.

Protein	Peptide Sequence	Test Method	Experimental Results (IC_50_)	References
Ovotransferrin	AGLAPYKLKPIA	*Escherichia coli* ATCC 25922 (Gram-negative bacteria) *Staphylococcus aureus* ATCC 25923 (Gram-positive bacteria) Minimal inhibitory concentration of peptide (MIC) values	32 μg/mL	[134]
Lysozyme	IVSDGDMNAW HGLDNYA	*Staphylococcus aureus* 23–394 (Gram-positive bacteria), *Escherichia coli* K-12 (Gram-negative bacteria) (MIC) values	400 μg/mL	[130]
	NTDGSTDYGILQINSR	*Escherichia coli* *Leuconostoc mesenteroides* bacteria (MIC) values	355.64 ± 2.2 μg/mL 442.25 ± 2.8 μg/mL	[40]
*Gallus gallus* apolipoprotein B	KGGDLGLFEPTL	*Staphylococcus aureus* ATCC29213 (MIC) values	2 mmol/L	[9]

**Table 5:** Other activities of HEPs.

Other Activities	Protein	Peptide Sequence	Test Method	Experimental Results	References
Anti-inflammatory activity	Ovotransferrin	IWHHT	The expression of vascular cell adhesion molecule-1 (VCAM-1)	IC_50_ μmol/L	[152]
		IRW IQW	Expression of adhesion molecules (ICAM-1 and VCAM-1)	Pretreatment with IRW (50 μmol/L) significantly inhibited the TNF-induced increased expression of both ICAM and VCAM-1 Pretreatment with IQW (50 μmol/L) significantly blocked the TNF-induced increased expression of ICAM-1	[138]
		GW	Inflammatory molecule expression (ICAM-1 and VCAM-1)	Reduction in TNF-α-induced VCAM-1 expression (64.3 ± 20.6%)	[153]
	Ovotransferrin	CR FL HC LL MK	Determination of IL-8	Reduction in IL-8	[154]
Immunomodulatory activity	Egg white protein	Egg white protein peptides (200 Da–500 Da)	Male BALB/c mice: The number of white blood cells in the peripheral blood Levels of serum cytokines	Supplementation with 750 mg/kg/d egg white protein peptides: Increase in IL-2, IL-6, TNF-α, and the number of white blood cells	[155]
Anti-cancer activity	Ovotransferrin	Ovotransferrin peptides prepared with promod 278P + thermolysin combination	MTT assay AGS LoVo HT-29 HeLa	IC_50_ (mg/mL) 0.79 0.78 0.92 0.78	[154]
Chelated minerals	Egg white protein	DHTKE	Calcium-binding capacity Binding sites of calcium on the DHTKE peptide	DHTKE: peptide = 1:1 The calcium-binding site corresponded to the carboxyl oxygen, amino nitrogen, and imidazole nitrogen atoms	[68]
		DKLPGFGDS(PO4)IEAQ	^59^Fe uptake in Caco-2 cells Ferritin expression in Caco-2 cells	Iron: synthetic peptide = 1:1, 80 ± 6.9% Increase in ferritin synthesis	[38]
	Phosvitin	DEEENDQVK	Calcium-binding capacity	151.1 mg Ca/g peptide	[67]
		EDDpSpS	Calcium-binding capacity	468 ± 2.80 mg Ca/g peptide	[32]

## Data Availability

No new data were created or analyzed in this study. Data sharing is not applicable to this article.

## Abbreviations

Hen Egg Proteins	HEPs
Phosvitin Phosphopeptides	PPPs
Egg White Protein	EWP
Ovalbumin	OVA
Ovotransferrin	OTF
Ovomucoid	OM
Ovomucin	OV
Lysozyme	LM
Phosvitin	PV
Immunoglobulin Y	IgY
Lysozyme Peptides	LMPs
Ovotransferrin-derived Peptides	OTFPs
Ovalbumin-derived Peptides	OVAPs
Pulsed Electric Field	PEF
Generally Regarded as Safe	GRAS
Molecular Weight Distribution	MWD
Ovomucin-derived Peptides	OMPs
Egg Yolk Peptides	EYPs
EWP Peptide	EWPP
Gastrointestinal	GI
Eggshell Membrane	ESM
Trolox Equivalents	TEs
High-temperature and Mild-pressure	HTMP
Lactic Acid Bacteria	LAB
Synthesis and Solid-Phase Synthesis	SSPS
Reverse Phase-High-Performance Liquid Chromatography	RP-HPLC
Mass Spectrometry	MS
Time Of Flight	TOF
Matrix-assisted Laser Deionization Time-of-flight	MALDI-TOF
Liquid Chromatography Triple Quadrupole Mass Spectrometry	LC-MS/MS
Electrospray Ionization Mass Spectrometry	ESI-MS
Triple Quadrupole Mass Spectrometer	TQ-MS
Quantitative Structure–Activity Relationship	QSAR
Reactive Oxygen Species	ROS
Cardiovascular Diseases	CVDs
Oxygen Radical Absorbance Capacity-Fluorescein	ORAC-FL
Ferric Ion-Reducing Antioxidant Potential	FRAP
2,2’-azino-bis 3-ethylbenzothiazoline-6-sulfonic acid	ABTS
2,2-diphenyl-1-picrylhydrazyl	DPPH
Gln-Ser-Leu-Val-Ser-Val-Pro-Gly-Met-Ser	QSLVSVPGMS
Angiotensin-I-Converting Enzyme	ACE, EC 3.4.15.1
Dipeptidyl Peptidase IV	DPP-IV, EC 3.4.14.5
Aminopeptidase N	APN, EC 3.4.11.2
Xanthine Oxidase	XO, EC 1.17.3.2,5
Renin–Angiotensin System	RAS
Kallikrein–kinin System	KKS
Gln-Ile-Gly-Leu-Phe	QIGLF
Phe-Glu-Ser-Asn-Thr-Gln-Ala-Thr-Asn-Arg	FESNFNTQATNR
Spontaneously Hypertensive Rats	SHRs
Nitric Oxide	NO
Dipeptidyl-peptidase IV Inhibitor	DPP-IVi
Glucagon-like Peptide-1	GLP-1
Glucose-dependent Insulinotropic Polypeptide	GIP
Ala-Asp-Phe	ADF
Met-Gly-Arg	MIR
Phe-Gly-Arg	FGR
Ile-Arg-Asp-Leu-Leu-Glu-Arg	IRDLLER
Try-Ala-Glu-Glu-Arg-Tyr-Pro	YAEERYP
Ile-Arg-Asn-Val-Leu-Gln-Pro-Ser	IRNVLQPS
Arg-Val-Pro-Ser-Leu-Met	RVPSLM
Cys-Asn-Arg	CNR
Cys-Asp-Arg	CDR
Cys-Glu-Phe	GEF
Lys-Ser-Trp-Lys-Lys-His-Val-Val-Ser-Gly-Phe-Phe-Leu-Arg	KSWKKHVVSGFFLR
Lys-Ser-Trp-Lys-Lys-His-Val-Val-Ser-Gly-Phe-Phe-Leu-Arg-Leu-Trp-Val-His-Lys-Lys	KSWKKHVVSGFFLRLWVHKK
Ovomucin Glycopeptide	OGP
Asn-Thr-Asp-Gly-Ser-Thr-Asp-Tyr-Gly-Ile-Leu-Gln-Ile-Asn-Ser-Arg	NTDGSTDYGILQINSR
Lys-Gly-Gly-Asp-Leu-Gly-Leu-Phe-Glu-Pro-Thr-Leu	KGGDLGLFEPTL
Mitogen-activated Protein Kinase	MAPK
Janus kinase-signal Transduction and Transcription Activator	JAK-STAT
Inducible Nitric Oxide Synthase	iNOS
IIe-Arg-Trp	IRW
IIe-Gln-Trp	IQW
Tumor Necrosis Factor	TNF
Vascular Cell Adhesion Molecule-I	VCAM-1
Asp-Gu-Gu-Gu-Asn-Asp-Gln-Val-Lys	DEEENDQVK
Thr-ASN-Gly-IIe-Arg	TNGIIR
Arg-Val-Pro-Ser-Leu	RVPSL
Gln-IIe-Gly-Leu-Phe	QIGLF
Leu-Gln-Lys	LQK
Leu-Leu	LL
Leu-Leu-Phe	LLF
Leu-Gln-Lys-Trp	LQKW
Angiotensin I	Ang I
Angiotensin II	Ang II
Angiotensin-Converting Enzyme 2	ACE2, EC 3.4.17.23
Renin-Angiotensin-Aldosterone System	RAAS
Alcalase	EC 3.4.21.62
Thermolysin	EC 3.4.24.27
Neutral proteases	EC 3.4.24.4
Protease N	EC 3.4.24.28
Protease A	EC 3.4.24.39
Trypsin	EC 3.4.21.4
Papain	EC 3.4.22.2
Pepsin	EC 3.4.23.1
α-chymotrypsin	EC 3.4.21.1
Chymotrypsin	232-671-2
Alkaline phosphatase	E.C.3.1.3.1
Neutrase	232-752-2
α-glucosidase	EC 3.2.1.1
Tyrosinase	EC 1.14.18.1
